# Characterization of the Infant Immune System and the Influence and Immunogenicity of BCG Vaccination in Infant and Adult Rhesus Macaques

**DOI:** 10.3389/fimmu.2021.754589

**Published:** 2021-10-11

**Authors:** Charlotte Sarfas, Andrew D. White, Laura Sibley, Alexandra L. Morrison, Jennie Gullick, Steve Lawrence, Mike J. Dennis, Philip D. Marsh, Helen A. Fletcher, Sally A. Sharpe

**Affiliations:** ^1^ National Infection Service, UK Health Security Agency, Salisbury, United Kingdom; ^2^ Department of Immunology and Infection, London School of Hygiene and Tropical Medicine, London, United Kingdom

**Keywords:** BCG, infant, immunology, macaque, infant vaccination, age comparison

## Abstract

In many countries where tuberculosis (TB) is endemic, the Bacillus Calmette–Guérin (BCG) vaccine is given as close to birth as possible to protect infants and children from severe forms of TB. However, BCG has variable efficacy and is not as effective against adult pulmonary TB. At present, most animal models used to study novel TB vaccine candidates rely on the use of adult animals. Human studies show that the infant immune system is different to that of an adult. Understanding how the phenotypic profile and functional ability of the immature host immune system compares to that of a mature adult, together with the subsequent BCG immune response, is critical to ensuring that new TB vaccines are tested in the most appropriate models. BCG-specific immune responses were detected in macaques vaccinated within a week of birth from six weeks after immunization indicating that neonatal macaques are able to generate a functional cellular response to the vaccine. However, the responses measured were significantly lower than those typically observed following BCG vaccination in adult rhesus macaques and infant profiles were skewed towards the activation and attraction of macrophages and monocytes and the synthesis in addition to release of pro-inflammatory cytokines such as IL-1, IL-6 and TNF-α. The frequency of specific immune cell populations changed significantly through the first three years of life as the infants developed into young adult macaques. Notably, the CD4:CD8 ratio significantly declined as the macaques aged due to a significant decrease in the proportion of CD4^+^ T-cells relative to a significant increase in CD8^+^ T-cells. Also, the frequency of both CD4^+^ and CD8^+^ T-cells expressing the memory marker CD95, and memory subset populations including effector memory, central memory and stem cell memory, increased significantly as animals matured. Infant macaques, vaccinated with BCG within a week of birth, possessed a significantly higher frequency of CD14^+^ classical monocytes and granulocytes which remained different throughout the first three years of life compared to unvaccinated age matched animals. These findings, along with the increase in monokines following vaccination in infants, may provide an insight into the mechanism by which vaccination with BCG is able to provide non-specific immunity against non-mycobacterial organisms.

## Introduction

Bacillus Calmette–Guérin (BCG) is one of the most widely used vaccines worldwide, yet the efficacy it confers is highly variable (0-80%) (1, 2) and how it affords protection from *Mycobacterium tuberculosis (M. tb)* is still not fully understood. In the UK, the BCG vaccine is only routinely given to those at high risk of tuberculosis (TB), but in endemic regions where the incidence of TB is high, such as parts of Africa and Asia, BCG vaccination is recommended as part of the neonatal vaccination programme, as it does afford protection from severe forms of the disease such as meningitis and miliary TB in children (2). BCG has been shown to improve the efficacy of other neonatal vaccines and has been linked to other beneficial effects resulting in reduced infant mortality (3, 4). Although the BCG vaccine is routinely administered to infants, there is little knowledge of the influence of BCG on the neonatal immune system.

The production of IFN-γ by effector cells in response to stimulation with tuberculin purified protein derivative (PPD) is often used as a measure of vaccine immunogenicity in clinical trials (5). Recent studies in non-human primates (NHPs) (6) have shown that increased frequency of IFN-γ secreting cells in response to PPD induced after vaccination significantly correlated with decreased disease burden and improved clinical parameters following challenge. A protective role for IFN-γ in the bovine model of TB has been suggested by Hope et al. (7), who showed increased levels of IFN-γ secreting memory T-cells post-vaccination in BCG vaccinated calves directly correlated with protection from disease and also, the magnitude of antigen-specific CD4^+^ IFN-γ cells post-challenge correlated with the severity of the disease.

There is currently no validated correlate of protection against *M. tb* infection and although IFN-γ is often used as a measure to assess vaccine immunogenicity, many studies have shown that IFN-γ, at least on its own, is not sufficient to provide protection (8). Other cytokines associated with the Th1 and Th17 response, such as TNF-α, IL-2 and IL-17, are thought to play an important role in protection from TB when co-expressed by multifunctional T-cells (9). Furthermore, memory cells that develop from T-cells with the capacity to produce multiple cytokines have the ability to provide more durable protection than cells producing IFN-γ alone (6, 10–13). IL-17 is also considered important in the regulation of the Th1 pro-inflammatory response (12, 14, 15).

In human new-borns, the ability to generate an effective inflammatory immune response to an antigen is greatly reduced compared to adults (3, 16, 17), as well as the capacity of CD4^+^ T-cells to produce IFN-γ, which is likely to affect the Th1 response induced by the vaccine (18). To prevent adverse immune reactions to maternal antigens, the fetal immune system is kept in a state of tolerance and is widely believed to be biased towards a Th2 response (19) with the pro-inflammatory Th1 response actively suppressed. In the period after birth, the neonate maintains partial tolerance to limit adverse immune reactions to antigens in the environment, and to allow colonization of commensal microorganisms which are beneficial to the host and help to prevent infection by pathogenic bacteria. Unfortunately, as infants have not yet developed a full complement of commensals, they are more prone to infections such as TB (20–22). As the fetus develops in a sterile environment and its pre-exposure to antigens and pathogens is minimal, to generate an effective response to a vaccination, a new-born must rely on its innate immune system rather than adaptive immune response to prevent infection.

There is evidence to suggest that human infants are able to mount an initial immune response following intradermal (ID) vaccination to BCG. A UK clinical trial (23) has identified a number of cytokines and chemokines involved in the generation, proliferation and recruitment of various cell subsets after BCG vaccination in infants. The molecules identified are involved in the Th1, Th2 and regulatory responses, and could provide biomarkers of protection when secreted together. Further, more recent studies in UK infants have described a BCG-induced, trained innate immunity response (24, 25).

The importance of understanding the infant immune response to vaccination is illustrated by the booster vaccine, MVA85A. When delivered to infants previously vaccinated with BCG, the IFN-γ response profiles measured using an ELISPOT assay were lower than in similarly boosted adults (26). As novel TB vaccines are likely to be given to individuals previously vaccinated at birth, pre-clinical models that accurately reflect the BCG primed infant immune system are vital to the development and identification of new, improved, vaccination strategies.

To date there have been few studies in which TB vaccines have been evaluated in models using neonatal and young animals. Prior to challenge with Simian Immunodeficiency Virus (SIV), BCG was delivered to infant NHPs within the first 2 weeks of life (27, 28). These studies demonstrated an initial immune response following intradermal (ID) vaccination with BCG in infancy (responses studied for up to nine weeks after BCG), with increases identified in the frequency, activation and function of CD4^+^ T-cells and also classical monocytes using flow cytometry and by gene expression analysis. Our study monitors changes in the macaque immune system following BCG vaccination given within a week of birth on the immune system during the first three years of life and directly compares the cellular immune response elicited by BCG in infants with that in similarly vaccinated mature adult macaques. Understanding the differences in the host response to BCG vaccination between the mature and immature immune systems (29) will be critical to the development and testing of vaccine strategies that aim to provide improved protection and enhanced immunogenicity, and, and which may facilitate the identification of correlates of protection and thereby further our knowledge of how BCG shapes the host immune system. This knowledge will inform decision making on future vaccines that may include vaccination in later in life with either a further dose of BCG or other boosting vaccines.

## Materials and Methods

### Experimental Animals

The animals used in these studies were male and female rhesus macaques (*Macaca mulatta*) of Indian genotype and obtained from an established, closed UK breeding colony (UKHSA, Porton Down, UK). Animals were housed in compatible social groups, in accordance with the Home Office (UK) Code of Practice for the Housing and Care of Animals Bred, Supplied or Used for Scientific Purposes (2014) and the National Committee for Refinement, Reduction and Replacement (NC3Rs) Guidelines on Primate Accommodation, Care and Use, August 2006. Enrichment was afforded by the provision of high-level observation balconies, swings, deep litter to allow foraging, feeding puzzles and toys. In addition to standard old-world primate pellets further food was provided by a selection of vegetables and fruit. Animals were sedated by intramuscular (IM) injection of ketamine hydrochloride (Ketaset, 100 mg/ml, Fort Dodge Animal Health Ltd, Southampton, UK; 10 mg/kg) for procedures requiring removal from their housing. Study design and procedures were approved by the UK Health Security Agency, Porton Down Animal Welfare and Ethical Review Body, and authorized under an appropriate UK Home Office project license.

**Figure 1:**
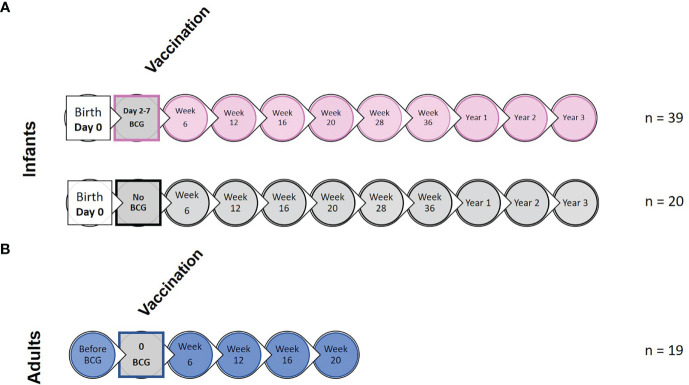
Study timeline relative to BCG vaccination and immunological analysis. **(A)** shows the study timeline in which infant rhesus macaques received BCG delivered by intradermal injection (n = 39) within a week of birth (two to seven days after birth) or remained unvaccinated as controls (n = 20). **(B)** Young adult macaques aged between 3.7 and 5.2 years of age received BCG delivered by intradermal injection at study week 0 (n = 19). Circles represent points relative to vaccination at which clinical examinations were conducted and blood samples collected for application of immunological analyses.

Sample collection and experimental design including application of vaccination is summarized in **Figure 1** and **Supplementary Figure 1**. Due to small volumes of blood permitted for collection from the infant macaques, animals were randomly assigned to sub-groups to enable completion of each type of immunological analysis

### Vaccination

Vaccinations were delivered as specified by the manufacturer’s guidelines and in accordance with current clinical protocols for application of BCG vaccination to human infants and adults (30). New-born rhesus macaques were immunized within seven days of birth with 50 μl Danish strain 1331 (Statens Serum Institute (SSI), Copenhagen, Denmark) delivered to the upper left arm by intradermal injection using a limited volume ‘insulin syringe’. Vaccination dose was verified by culture of the residual vaccine on Middlebrook 7H11 selective agar (between 1-6 x 10^6^ CFU/ml, data not shown). Adult macaques were vaccinated in an identical fashion but with 100 μl of BCG Danish strain 1331 as specified by the manufacturer’s guidelines.

### Blood Sample Collection

Macaques were sedated at regular intervals for blood sample collection. For welfare reasons, infant macaques were not sedated during blood sample collection until they reached an age where their activities were comparatively independent of the mother and of a size where safe restraint was impracticable. Blood was collected from the femoral vein using a needle and syringe and dispensed into heparin tubes (Sigma Aldrich, Gillingham, UK) for use in immunological analysis. The volume of blood taken was calculated in line with body weight and in accordance with the recommendations of the BVA/FRAME/RSPCA/EFAW Joint Working Group on Refinement Recommendations for Removal of Blood from Laboratory Animals (31).

Body weight, body temperature and red blood cell (RBC) hemoglobin levels were measured at each time point. RBC hemoglobin concentration was measured using a HaemaCue haemoglobinometer (Haemacue Ltd, Dronfield, UK) and erythrocyte sedimentation rate (ESR) was measured using the Sediplast system (Guest Medical, Edenbridge, UK). Animal behavior was observed daily throughout the study.

### Immune Response Analysis

#### Interferon-Gamma (IFN-γ) ELISPOT

ELISPOT assays were performed on peripheral blood mononuclear cells (PBMC) isolated from heparin anti-coagulated blood to measure the frequency of cells producing Interferon-gamma (IFN-γ) in response to stimulation with 10 µg/ml purified protein derivative (PPD) (SSI, Copenhagen, Denmark) using standard methods, as previously described (6).

### Quantification of Secreted Biomarkers in Mycobacterial Antigen Stimulated Blood Cultures

Antigen-specific secretion of a range of cytokines, chemokines and growth factors was assessed by dilution of heparinized blood samples 1:10 with serum free Roswell Park Memorial Institute (RPMI) (R0) medium supplemented with L-glutamine (2 mM), penicillin (50 U/ml) streptomycin (50 μg/ml) (all from Sigma Aldrich, Gillingham, UK), before six day culture at 37°C, 5% CO2, with 10 µg/ml tuberculin PPD (Statens Serum Institute, Copenhagen, Denmark), or 10 µg/ml phytohaemmagglutinin (PHA) from *Phaseolus vulgaris* (Sigma Aldrich, Gillingham, UK) as positive control, or R0 alone as a negative control. Following incubation, culture supernatants were aspirated using a one ml syringe (BD Biosciences, Oxford, UK) and passed through two 0.2 µm polyether sulfone (PES) membrane filters (GE Life Sciences, Amersham, UK), before storage at -80°C. Secreted biomarkers in filtered culture supernatants were quantified using a 37-plex Procartaplex bead array assay (Thermo Fischer Scientific, UK) applied according to the manufacturer’s instructions for detection of the following analytes: Eotaxin, G-CSF, GM-CSF, IFN-alpha, IFN-gamma, TNF-alpha, IL-10, IL-12p70, IL-13, IL-15, IL-17A, IL-18, IL-1b, IL-1RA, IL-2, IL-23, IL-4, IL-5, IL-6, IL-7, IL-8, IP-10, I-TAC (CXCL11), MCP-1 (CCL2), macrophage inflammatory protein-1α (MIP-1alpha), macrophage inflammatory protein-1α (MIP-1beta) (CCL4), SDF-1alpha (CXCL12), MIG (CXCL9), CD40-Ligand, BLC (CXCL13), BDNF, SCF, VEGF-D, bNGF, FGF-2, PDGF-BB, VEGF-A. Culture filtrates were analyzed using a Luminex Magpix instrument (Luminex Corporation, US) equipped with the xPONENT 4.2 software package (Luminex Corporation). Standard curves were generated in duplicate and used to interpolate the concentration of each analyte. All data below the limit of detection specified by the kit manufacturer were assigned a zero value. The analyte concentration measured in negative control cultures was subtracted from antigen stimulated samples to obtain a measure of antigen-specific biomarker secretion and the analyte concentration was multiplied by ten to account for the initial dilution of the sample and provide a value per ml of blood.

### Polyfunctional Intracellular Cytokine Staining and Antigen-Specific Memory T-Cell Assay

Intracellular cytokine staining (ICS) was performed using 1 x 10^6^ PBMC in medium (R10) consisting of RPMI 1640 supplemented with L-glutamine (2 mM), penicillin (50 U/ml) streptomycin (50 μg/ml) (all from Sigma Aldrich, Gillingham, UK) and 10% heat-inactivated foetal bovine serum (Labtech Ltd, Uckfield, UK). These cells were stimulated with a 10 µg/ml solution of CD28 and CD49d co-stimulatory antibodies (both from BD Biosciences, Oxford, UK) and either 10 µg/ml PPD (SSI, Copenhagen, Denmark), or 5 µg/ml staphylococcal enterotoxin b (SEB) (Sigma Aldrich, Gillingham, UK), or R10 medium alone as negative control, for a total of six hours at 37°C, in a 5% CO_2_ supplemented incubator. Following the initial two hours of incubation, the protein transport inhibitor Brefeldin-A (Sigma Aldrich, Gillingham, UK) was added to the incubation mixture at a final concentration of 10 µg/ml. Following incubation, cells were washed by centrifugation at 400g for 5 minutes with FACS buffer consisting of PBS + 1% FCS and incubated for 30 minutes at room temperature with optimal dilutions of the amine-reactive Live/Dead Fixable Red viability cell stain (Life Technologies, Renfrew, UK) and the antibodies CD4 PerCP-Cy5.5 (BD Biosciences, Oxford, UK), CD8 BV510, CD28 BV421, CD95 Pe-Cy7, CD56 BV605, CD16 BV785, CD14 PeDazzle594, CD20 PeDazzle594 (all Biolegend, London, UK), CCR7 PE (eBioscience, Hatfield, UK), CD45RA APC-Vio770, (both Miltenyi Biotech Ltd, Bisley, UK). Following surface marker staining, the cells were washed and then permeabilized by incubation at room temperature for 15 minutes with Fix/Perm reagent (BD Biosciences, Oxford, UK). Further cell washes were applied by centrifugation using Permwash buffer (BD Biosciences, Oxford, UK), before staining for intracellular antigens by incubation at room temperature for 30 minutes with the antibodies CD3 AF700, IFN-γ FITC, TNF-α BUV395 (all from BD Biosciences, Oxford, UK), IL-2 APC (Miltenyi Biotech Ltd, Bisley, UK), IL-17 BV711 (Biolegend, London, UK).

Unstimulated PBMCs were stained with CD4 PerCP-Cy5.5, CD25 FITC (BD Biosciences, Oxford, UK), CD8 BV510, CD14 PeDazzle594, CD20 PeDazzle594 (Biolegend, London, UK), CD127 APC (eBioscience, Hatfield, UK) to investigate CD4^+^ T-regulatory cells.

BD Compbeads (BD Biosciences, Oxford, UK) were labelled with the above fluorochromes for use as compensation controls. Following incubation with the antibodies for 30 mins in the dark at room temperature, cells and beads were washed by centrifugation at 400g for 5 minutes and fixed in 4% paraformaldehyde solution (Sigma Aldrich, Gillingham, UK) prior to flow cytometric acquisition.

### Measurement of Host Cell Status Using Whole Blood Intracellular Cytokine Staining

Freshly collected blood anti-coagulated with heparin was incubated with a solution of 0.1 µg/ml of CD28 and 0.1 µg/ml of CD49d costimulatory antibodies (BD, Biosciences, Oxford, UK) alone (negative control), or with PPD (10 µg/ml), or SEB (5 µg/ml) (positive control) (Sigma Aldrich, Gillingham, UK) at 37°C for 5 hours, then a further 16 hours with Brefeldin A (10ug/mL) (Sigma Aldrich, Gillingham, UK). After incubation, 40 µl of 20 mM EDTA (Ethylenediaminetetraacetic acid solution) (Sigma Aldrich, Gillingham, UK) was added to remove adherent cells and after 15 minutes, red blood cells were lysed using FACS Lysing Solution (BD, Biosciences, Oxford, UK). Cells were washed by centrifugation at 400g for five minutes then stored in cryomedium consisting of heat inactivated FBS (Labtech Ltd, Uckfield, UK) with 10% dimethyl sulfoxide (DMSO) (Sigma Aldrich, Gillingham, UK) at -196°C until required for analysis.

Following resuscitation, cells were washed by centrifugation in FACS buffer and then incubated for 30 minutes at room temperature with fluorescent antibodies: CD4 APC-H7, CD8 PerCPCy5.5, CD16 BV421 (all from BD Biosciences, Oxford, UK), CD14 PE, CD20 ECD (all from Beckman Coulter, High Wycombe, UK), CD95 BV605, HLA-DR BV785, (all from Biolegend, London, UK) in 50 µl BD Brilliant stain buffer (BD Biosciences, Oxford, UK). Cells were washed by centrifugation at 400g for 5 minutes before permeabilization in cytoperm/cytofix solution (BD Biosciences, Oxford, UK) for 15 minutes, washed by centrifugation at 400g for 5 minutes with Perm/wash buffer (BD Biosciences, Oxford, UK) before staining with fluorescent antibodies: CD3 AF700, IFN-γ FITC, TNF-α BUV395 (all from BD Biosciences, Oxford, UK), IL-17 BV711 (Biolegend, London, UK), IL-2 APC, (Miltenyi Biotech, Bisley, UK), in 50 µl BD Brilliant stain buffer (BD Biosciences, Oxford, UK) for 30 minutes at room temperature. Incubation was followed by washing of the cells by centrifugation as described previously with Perm/wash buffer (BD Biosciences, Oxford, UK) prior to fixing in 4% paraformaldehyde (Sigma Aldrich, Gillingham, UK). Cells were analyzed using flow cytometry. BD Compbeads (BD Biosciences, Oxford, UK) were labelled with matching fluorochromes and used as compensation controls.

### Flow Cytometric Acquisition and Analysis

Cells were analyzed using a five laser LSRII Fortessa instrument (BD Biosciences, Oxford, UK) and data were analyzed using FlowJo (version 9.7.6, Treestar, Ashland, US). Cytokine-producing T-cells were identified using a forward scatter-height (FSC-H) *versus* side scatter-area (SSC-A) dot plot to identify the lymphocyte population, to which appropriate gating strategies were applied to exclude doublet events, non-viable cells, monocytes (CD14^+^) and B cells (CD20^+^). For ICS analysis, sequential gating through CD3^+^, followed by CD4^+^ or CD8^+^ gates were used before individual cytokine gates to identify IFN-γ, IL-2, TNF-α and IL-17 producing populations. Polyfunctional cells were identified using Boolean gating combinations of individual cytokine-producing CD4 or CD8 T-cells. Antigen-specific T-cell memory profiles were identified by applying a summed CD4 or CD8 cytokine Boolean combination, followed by gating for CD95 surface staining. Differentiation of effector, transitional effector, central memory, and stem cell memory T-cell populations was established by CD45RA, CD28 and CCR7 expression patterns. The software package PESTLE version 1.7 (Mario Roederer, Vaccine Research Centre, NIAID, NIH) was used for background subtraction to obtain antigen-specific polyfunctional ICS and memory T-cell cytokine responses, Graphpad Prism (version 8.0.1) was used to generate graphical representations of flow cytometry data.

### Statistical Analyses

Data from IFN-γ ELISPOT assay, multiplex cytokine release assay and both phenotypic and multiparameter flow cytometry assays were analyzed using GraphPad Prism Software (version 8.0 1 La Jolla, California, USA). Age-specific differences of immune cell populations and T-cell functionality, both before and after vaccination, were compared between each age group using a non-parametric Mann-Whitney U-test. Similarly, vaccine-induced changes in both cell frequency and T-cell functional profiles were compared within the vaccination groups using a Wilcoxon-rank test. Statistical significance was defined as a p-value equal to or lower than 0.05.

The software package PESTLE (version 1.8) was used for background subtraction to determine antigen-specific ICS responses. Graphical representation and statistical analysis of the data was performed using GraphPad Prism Software (version 8.0 1 La Jolla, California, USA). Negative values in functional data generated by background subtraction were assigned a zero value.

## Results

### Antigen-Specific Cellular Immune Responses Measured Following BCG Vaccination

#### Frequency of PPD-Specific IFN-γ Secreting Cells Measured by ELISPOT Assay

**Figure 2:**
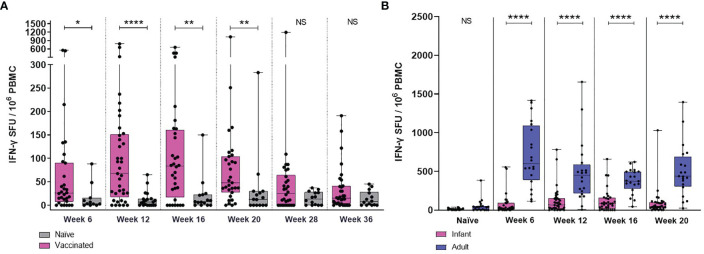
PPD-specific IFN-γ spot forming unit (SFU) frequency measured by ELISPOT following ID BCG vaccination. **(A)** IFN-γ-specific SFU from BCG vaccinated macaques and unvaccinated macaques during the initial 36 weeks after vaccination. **(B)** IFN-γ-specific SFU from BCG vaccinated infant (pink) and young adult (blue) macaques during the first 20 weeks after vaccination. Box plots show the group median +/- the inter-quartile range measured at each time point after vaccination, with minimum and maximum values connected by whiskers. Significant differences measured by Mann-Whitney U-test between the groups are indicated. *p ≤ 0.05, **p < 0.01, ****p < 0.0001, NS, not significant. n=39 for BCG vaccinated (pink), n=20 for unvaccinated controls (grey) and n=19 for young adults (blue).

The frequency of PPD-specific IFN-γ secreting cells was evaluated in macaques vaccinated within a week of birth and compared to unvaccinated age-matched controls. Samples were taken at regular intervals from six weeks until 36 weeks of age. Responses measured in BCG vaccinated and unvaccinated age-matched macaques were compared at each time point. BCG vaccinated macaques possessed a significantly higher frequency of PPD-specific IFN-γ secreting cells at weeks six (*p =* 0.0203), 12 (*p =* 0.0001), 16 (*p =* 0.0093) and 20 (*p =* 0.0012) than those who did not receive the vaccine. The level of response was not significantly different between the vaccination groups at week 28 and 36 indicating that the frequency of PPD-specific IFN-γ secreting cells had returned to a level equivalent to unvaccinated control animals (**Figure 2A**). The young adult animals had a significantly higher frequency of antigen-specific IFN-γ secreting cells at all time points following BCG vaccination compared to those vaccinated at birth (p < 0.0001) (**Figure 2B**).

### Secretion of PPD-Specific Biomarkers Measured by Cytokine Bead Array

**Figure 3:**
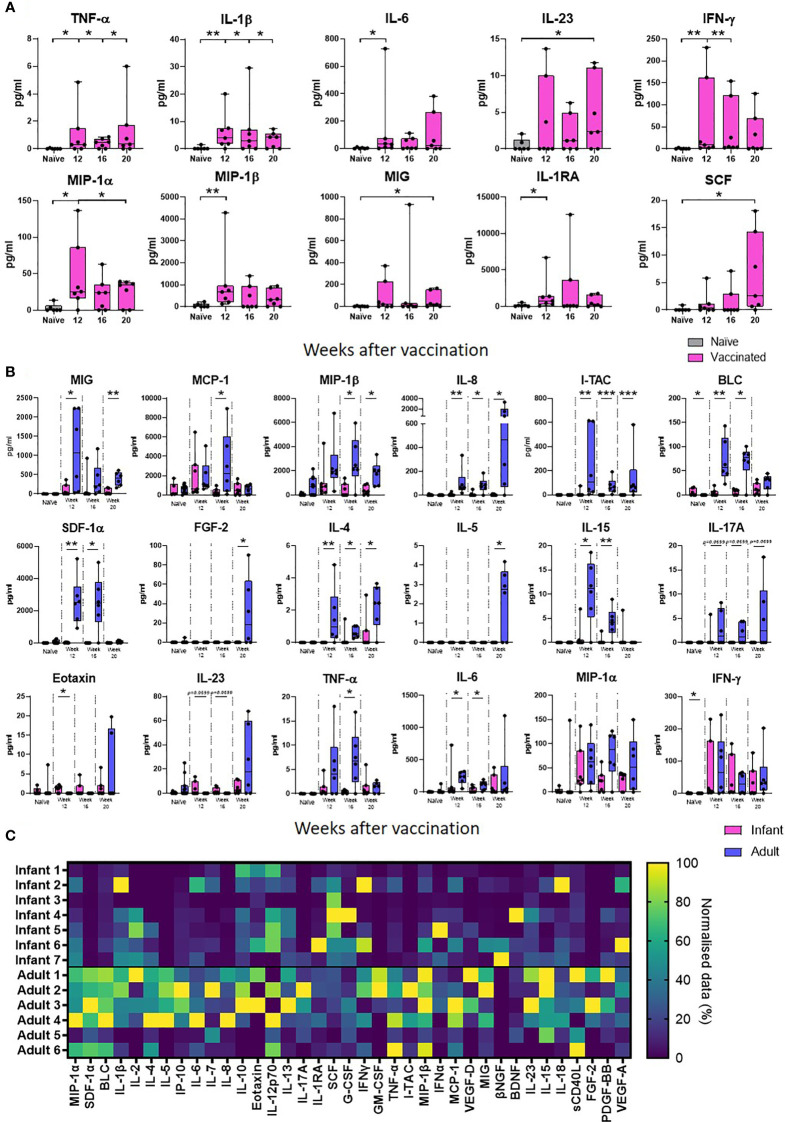
PPD-specific secretion of cytokine, chemokine and growth factor biomarkers measured in whole blood culture supernatant. Box plots **(A)** show the group median titre of each cytokine, chemokine or growth factor +/- the inter-quartile range measured in naïve (grey) infant macaques or at weeks 12, 16 and 20 after vaccination (pink), with minimum and maximum values connected by whiskers. Box plots **(B)** show the group median titre of each cytokine, chemokine or growth factor +/- the inter-quartile range measured in naïve infant macaques (pink) or naïve adult macaques (blue) at 12, 16 and 20 weeks after vaccination in each age group, with minimum and maximum values connected by whiskers. Dots represent individual animals. Significant differences measured by Mann-Whitney U-test between the groups are indicated. *p ≤ 0.05, **p < 0.01, ***p < 0.001. **(C)**. Quantity of PPD-specific secretion of cytokine, chemokine and growth factor biomarkers between weeks 12 and 20 (AUC) in stimulated supernatants in macaques vaccinated as infants and adults in a heat map plot. Data was normalized to give a proportional (%) representation of AUC values calculated from the profile of each analyte relative to the maximal amount of each analyte measured.

Supernatants from PPD-stimulated cultures of whole blood collected at 12, 16 and 20 weeks after neonatal vaccination with BCG from seven infant macaques were evaluated for the presence of 37 cytokines, chemokines and growth factors to identify vaccine-specific responses and compared that of unvaccinated animals. Due to limited blood volumes permissible for collection from the infant animals, samples were not available for the unvaccinated animals at each equivalent time point to the BCG vaccinated infants. An average was taken of the two earliest time points available from unvaccinated infant macaques (all before 20 weeks of age). Of the 37 analytes measured, the concentration of 27 (IL-2, IL-4, IL-5, BLC, IL-7, IL-8, IL-10, IP-10, MCP-1, SDF-1α, Eotaxin, IL-12p70, IL-13, IL-17A, IL-18, sCD40L, G-CSF, GM-CSF, I-TAC, IFN- α, IL-15, VEGF-D, βNGF, BDNF, PDGF-BB, FGF-2 and VEGF-A) were either undetectable (below the limit of detection of the assay system), or did not differ from the unvaccinated age-matched controls following BCG vaccination. The concentrations of the remaining ten analytes were significantly higher in the group that received a BCG vaccination in comparison to unvaccinated age-matched controls. Secretion of the pro-inflammatory cytokine TNF-α was significantly higher at weeks 12, 16 and 20 (*p =* 0.0332 for all time points) than in macaques that had not received BCG (naïve). The BCG vaccinated group also had significantly higher concentrations of IL-1 at week 12 (*p =* 0.0064), week 16 (*p =* 0.0495) and week 20 (*p =* 0.0495); IL-6 at week 12 (*p =* 0.0216); IL-23 at week 20 (*p =* 0.05); and IFN-γ at week 12 (*p =* 0.0047) and week 16 (*p =* 0.0023). Chemokine markers MIP-1α, MIP-1β and MIG which are typically produced by macrophages and monocytes were significantly increased in BCG vaccinated macaques compared to unvaccinated age matched controls at 12 weeks (MIP-1α: *p =* 0.0222, MIP-1β: *p =* 0.0047) and 20 weeks (MIP-1α: *p =* 0.05, MIG: *p =* 0.05). Modulatory cytokines IL-1RA and SCF were also significantly increased in the vaccinated group at week 12 (*p =* 0.0167) and week 20 (*p =* 0.0216), respectively (**Figure 3A**).

The concentration of analyte secreted in response to PPD-stimulation measured after BCG vaccination in infants was compared to that secreted into whole blood cultures prepared from similarly vaccinated adult macaques at weeks 12, 16 and 20 after receiving the BCG vaccine. The concentration of cytokine produced in response to antigenic stimulation in samples collected before vaccination in the adult group was compared to the concentration secreted by unvaccinated infant macaques. As before, the presence of 37 cytokines, chemokines and growth factors to identify vaccine-specific responses were evaluated and compared. Of the 37 analytes measured, the concentration of 24 cytokines, chemokines and growth factors were found to significantly increase after BCG was given to adult macaques. Of the ten analytes that were significantly increased after vaccination in the infant vaccinated group, the concentrations of four IL-1β, IL-23 SCF and Eotaxin were uniquely found to be significantly higher after BCG vaccination in the infant group only, whereas 19 analytes; MCP-1, MIP-1β, I-TAC, SDF-1α, BLC, IL-2, IL-4, IL-5, IL-7, IL-8, IL-10, IP-10, IL-12p70, IL-13, IL-15, IL-17A, IL-18, VEGF-D, PDGF-BB and FGF-2 were significantly increased after vaccination in the adult vaccinated group only. PPD-specific secretion of eight chemokines; MIG, MCP-1, MIP-1β, IL-8, I-TAC, SDF-1α and BLC was found to be significantly higher following BCG vaccination in the adult group compared to the infant group (*p* < 0.05). The concentrations of growth factor, FGF-2 and cytokines IL-4, IL-5, IL-15 and TNF-α were also found to be significantly higher (*p* < 0.05 or close to significance, *p =* 0.0699, for IL-17A) after vaccination in the adult group. There was a trend (*p =* 0.0699) for IL-23 to be increased at both week 12 and 16 after BCG in the infant vaccinated group compared to the adult group. Eotaxin was detected at significantly higher levels at week 12 after vaccination (*p =* 0.0210) in the macaques vaccinated within a week of birth. Both the macaques vaccinated in infancy and those vaccinated in adulthood showed significantly higher concentrations of IL-6, MIG, MIP-1α, MIP-1β, TNF-α and IFN-γ after BCG vaccination. However, IL-6, MIG, MIP-1β and TNF-α were detected at significantly higher concentrations in the adult vaccinated group (**Figure 3B**).

To evaluate the total amount of PPD-specific secretion of cytokine, chemokine and growth factor biomarkers after BCG vaccination in macaques vaccinated within a week of birth or as adults, the area under the curve (AUC) between weeks 12 and 20 after vaccination was calculated and represented as a heatmap (**Figure 3C**). More analytes were detected at a higher concentration in the adult vaccinated animals compared to those vaccinated in infancy. These included both pro- and anti-inflammatory chemokines and cytokines. Vaccinated infants secreted higher concentrations of immunomodulatory cytokines IL-1RA and SCF.

### Age Related Change in Lymphocyte and Memory T-Cell Populations

Lymphocyte population and subset frequencies were characterised in macaques after BCG vaccination and in naïve age-matched controls, to determine the impact of age on T cell status and the evolution of T-cell memory. Immunophenotyping was applied to cryopreserved PBMC collected at regular intervals from nine naïve macaques between the age of six weeks and three years along with PBMC from eight naïve macaques age four to five years of age to determine the frequency of CD3^+^, CD4^+^, CD8^+^ and CD4^+^CD8^+^ T-cells and also CD4^+^ and CD8^+^ memory T-cell subpopulations. Memory cells were identified by their phenotypic markers by gating on either the CD4^+^ or CD8^+^ populations with high surface staining for CD95. Central and effector memory T-cells were identified by their differential expression patterns of the co-stimulatory receptor CD28 and lymph node homing marker CCR7 (32, 33). T-cell memory phenotype was determined on CD95^+^ cells as CD28^+^CCR7^+^ central memory (TCM), CD28^+^CCR7^−^ transitional effector memory (TEM1) and effector memory (TEM2) CD28^-^CCR7^−^. Stem cell memory CD28^+^CCR7^+^ CD45RA^+^ (TSCM) and terminal effector memory cells CD28^-^CCR7^-^ CD45RA^+^ (TTE) were also investigated (**Supplementary Figure 2**).

The frequency of CD3^+^ cells did not significantly change as the macaques matured through the first three years of life. However, the group of young adult macaques had a significantly lower frequency of CD3^+^ T-cells compared to that measured in the longitudinal study cohort at six weeks of age.

**Figure 4:**
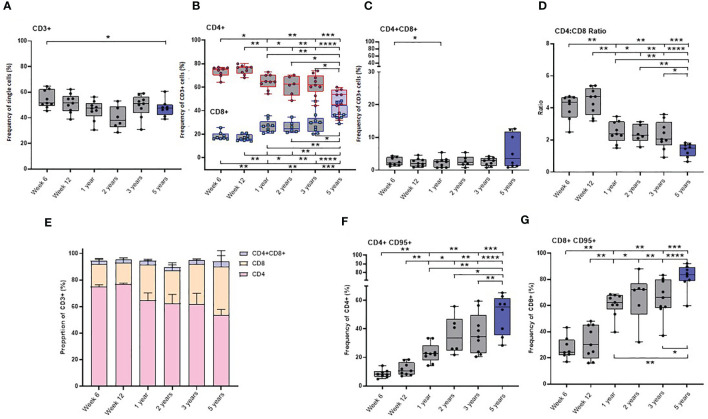
Age related changes in lymphocyte and memory cell subset populations. Plots **(A–D)** show the frequency of lymphocytes subsets, **(E)** proportions of CD4+, CD8+ and CD4+CD8+ lymphocytes **(F)** CD4+CD95+ and **(G)** CD8+CD95+ memory cells measured in naïve macaques from infancy (6 weeks of age) through to three years (grey) compared with young adult macaques aged between 3.7 and 5.2 years of age (blue). Box plots show the group median +/- the inter-quartile range, with minimum and maximum values connected by whiskers. Significant differences measured by Wilcoxon matched-pairs rank test or Mann-Whitney U-test between the groups are indicated. *p < 0.05, **p < 0.01, ***p < 0.001, ****p < 0.0001.

As naïve new-born macaques progressed through the first 12 weeks of life, the frequency of both CD4^+^ and CD8^+^ T-cells remained stable. A significant decline in the CD4:CD8 ratio occurred during the first year of life (*p =* 0.0078), due to a significant decrease in CD4^+^ T-cells and a significant increase in CD8^+^ T-cells, but thereafter remained stable throughout adolescence to three years of age. The CD4:CD8 ratio measured in the young adult group was significantly lower than that measured in the cohort of growing macaques at week six (*p =* 0.0002), week 12 (*p <*0.0001), one year (*p =* 0.0037), two years (*p =* 0.0047) and three years (*p =* 0.0206) which suggests that the cell populations present in the host continue to change as macaques age (**Figures 4A–E**).

The CD4^+^ and CD8^+^ T-cells present in infants were found to have a predominantly naïve phenotype lacking the memory marker CD95. Expression of the FAS signaling receptor CD95, a common marker of T-cell maturation/memory status (34) did not change between six and 12 weeks of age. Subsequently, the frequency of CD95 expressing memory cells significantly increased in both the CD4^+^ and CD8^+^ T-cell populations (**Supplementary table 1**) over the first five years of life. (**Figures 4F, G**).

The frequency of almost all CD4^+^ and CD8^+^ memory subsets (Supplementary **Figures 3A–L**) significantly increased as the macaques aged. The frequency of CD8^+^ TSCM, measured in the cohort of growing macaques over the first three years of life was significantly lower than that measured in the older group of young adults (comparison to six week old: *p =* 0.0019; 12 week old: *p =* 0.0055, one-year old: *p =* 0.0016; two-year-old: *p =* 0.02) (**Supplementary Figure 3K**). Only low levels of CD4^+^ effector memory (TEM2) cells and terminally differentiated (TTE) cells were present in infant macaques at six or 12 weeks of age (**Supplementary Figures 3I, J**).

Immunophenotyping was also applied to PBMCs collected from seven macaques vaccinated within a week of birth in order to investigate the impact of BCG on the evolution of lymphocytes and T-cell memory. Different memory cell subsets, including effector memory and central memory T-cell subsets were compared. The frequency of CD8^+^ TTE cells was found to be significantly higher in the unvaccinated macaques at two years of age compared to those who were vaccinated in infancy (*p =* 0.0260) (**Supplementary Figure 4P**). BCG did not affect the frequencies of lymphocytes or other memory cell subsets present as macaques aged (**Supplementary Figure 4**).

**Figure 5:**
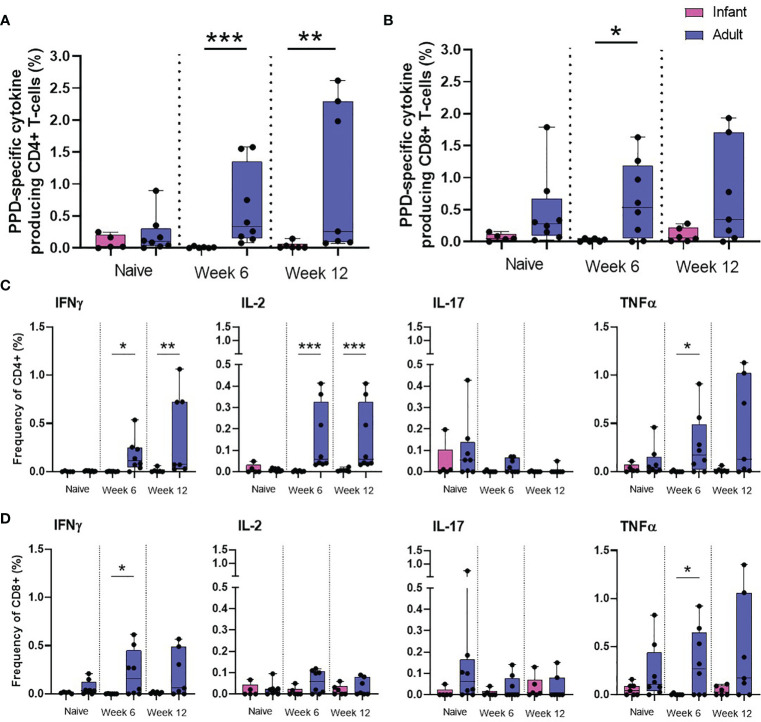
PPD-specific cytokine production by CD4 and CD8 memory T-cells. Total PPD-specific cytokine production by CD4+ and CD8+ T-cells. Dots represent the summed frequency of CD4+ **(A)** and CD8+ **(B)** T-cells producing IFN-γ, IL-2, TNF-α and IL-17 and CD4+ **(C)** and CD8+ **(D)** T-cells producing cytokines IFN-γ, IL-2, TNF-α and IL-17 measured in PBMCs collected from individual infant (pink) and adult (blue) animals prior to and following ID BCG vaccination. Box plots show the group median +/- the inter-quartile range, with minimum and maximum values connected by whiskers. Significant differences measured by Mann-Whitney U-test between the groups are indicated. *p ≤ 0.05, **p < 0.01, ***p < 0.001.

To further investigate the impact of age on the immune response elicited by BCG on memory cell populations, multi-parameter ICS staining was applied to PBMCs collected from the seven macaques vaccinated within a week of birth, nine unvaccinated infant age matched controls and eight young adult animals. The frequency of cells producing the cytokines IFN-γ, TNF-α, IL-2 and IL-17 were measured just prior to BCG vaccination (week 0) and at week six and 12 after vaccination in the young adult macaque group to provide a direct comparison to the macaques vaccinated within a week of birth. Due to the age at vaccination, the collection of blood samples prior to BCG was not possible for the neonatally vaccinated group, therefore, the first samples collected from unvaccinated macaques at six weeks of age were used to represent the immune responses in infants before vaccination. There was significantly more cytokine produced by CD4^+^ memory (CD95^+^) cells at week six (*p =* 0.0007) and week 12 (*p =* 0.0082) and also at week six (*p =* 0.0310) in the CD8^+^ memory cells in the macaques vaccinated as young adults compared to those vaccinated within a week of birth (**Figures 5A, B**). The frequency of CD4^+^ cells producing IFN-γ (week six: *p =* 0.0130, week 12: *p =* 0.0047), IL-2 (week six: *p =* 0.0007, week 12: *p =* 0.0007) and TNF-α (week six: *p =* 0.0210) in BCG vaccinated adults was significantly higher than in infants. The frequency of CD8^+^ cells producing IFN-γ (week six: *p =* 0.0210) and TNF-α (week six: *p =* 0.05) in BCG vaccinated adults was significantly higher than in infants (**Figures 5C, D**).

### Age Related Changes in Composition of the Cellular Immune Compartment in Whole Blood

The frequency of white cell populations in the peripheral blood of six naïve neonatal macaques was assessed by immunophenotyping applied at the earliest opportunity that sufficient blood volume could be collected (12 weeks of age), then at 16 weeks and 20 weeks of age, then yearly until the macaques reached three years of age. Population frequencies were compared to those measured in 14 young adult macaques aged between 3.7 and 5.2 years to identify age-associated changes in the host.

**Figure 6:**
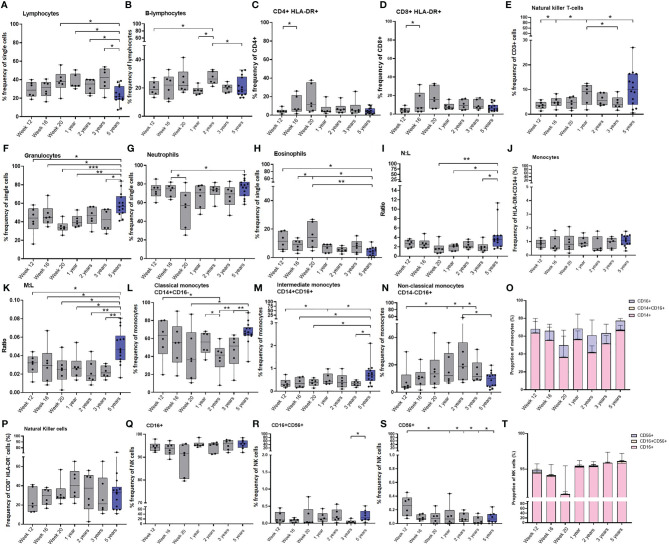
Age related changes in white cell populations measured in whole blood. Plots show the frequency of **(A)** All lymphocytes, **(B)** B-cells, **(C)** activated CD4+ and **(D)** CD8+ T-cells, **(E)** NK T-cells, **(F)** Granulocytes, **(G)** Neutrophils, **(H)** Eosinophils, **(I)** Neutrophil: Lymphocyte (N:L) ratio, **(J)** All monocytes, **(K)** Monocyte: Lymphocyte (M:L) ratio, **(L)** Classical monocytes **(M)** Intermediate monocytes and **(N)** Non-classical monocytes. **(O)** Bars represent median values of monocyte subset populations. **(P)** Natural killer cells, **(Q)** CD16+ NK cells, **(R)** CD16+CD56+ NK cells, **(S)** CD16-CD56+ NK cells and **(T)** Bars represent median values of NK cell subsets. Cell frequency populations were measured in naïve macaques from infancy through to three years (grey) compared with young adult macaques aged 3.7 and 5.2 years of age (blue). Box plots show the group median +/- the inter-quartile range, with minimum and maximum values connected by whiskers. Significant differences measured by Wilcoxon matched-pairs rank test or Mann-Whitney U-test between the groups are indicated. *p ≤ 0.05, **p < 0.01, ***p < 0.001.

T and B-cells were identified by their size and granularity scattering properties using a forward scatter-area *versus* side scatter-area dot plot, to which an appropriate step by step gating strategy was applied to exclude doublet cells. B-cells, T-cells, CD4^+^ and CD8^+^ T-cells were identified by their expression of CD20, CD3, CD4 and CD8 markers respectively (**Supplementary Figure 5**). In line with the results of intracellular cytokine staining studies of PBMCs described above, the frequency of the CD3^+^ lymphocyte population did not change and a significant decline in the CD4:CD8 ratio was identified as macaques matured (data not shown). The frequency of total lymphocytes did not change over the first three years of life. However, young adult macaques had a significantly lower number than macaques aged 20 weeks (p = 0.0154), one (p = 0.0154), two (p = 0.0247) and three (p = 0.0117) years of age (**Figure 6A**). The frequency of the B-cell population remained relatively consistent as the new-born macaques progressed through the first 20 weeks of life, and as animals matured over the first 3 years of life levels aligned with those determined in the group of young adult males. However, a peak in cell numbers was identified at two years of age when levels reached a significantly higher frequency than those seen at 12 weeks after birth (*p =* 0.0313), one year (*p =* 0.0313), and almost reached significance at three years of age (*p =* 0.0625) as animals matured. Similarly, the frequencies recorded at two years of age were significantly greater than those measured in the young adult macaque group (*p =* 0.0487) (**Figure 6B**). Activated T-cells were identified by their phenotypic markers by gating on either the CD4^+^ or CD8^+^ populations with high surface staining for activation marker HLA-DR (**Supplementary Figure 5**). A significant increase between weeks 12 and 16 (*p =* 0.0313), followed by a continued trend for an increase between weeks 16 and 20 (*p =* 0.0625) in the proportion of CD4^+^ and CD8^+^ cells expressing the activation marker HLA-DR occurred during the first 5 months of life, potentially due to contact with environmental bacteria and viruses such as mycobacteria and cytomegalovirus (CMV) (35–37) (**Figures 6C, D**). Thereafter, the frequency of cells expressing activation marker HLA-DR did not change significantly as macaques progressed from infancy to early adulthood. Natural killer (NK) T-cells were identified by gating on the CD3 population that also expressed surface marker CD16 (**Supplementary Figure 5**). The frequency of NK T-cells significantly increased between the age of week 12 and 16 (*p =* 0.0313) and at one year of age (*p =* 0.0313). There were significantly more NK T-cells present in young adults compared to the level present at 12 weeks of age (*p =* 0.05). In contrast to the number of NK T-cells detected which declined between the age of 1 and 3 years (*p =* 0.0313) (**Figure 6E**).

Granulocytes were identified by their size and granularity scattering properties using a FSC-A *vs* SSC-dot plot with eosinophils and neutrophils differentiated by expression of CD14 and HLA-DR (**Supplementary Figure 6**). As the new-born macaques progressed through the first 20 weeks of life, the frequency of granulocytes remained consistent. However, within the granulocyte population, the frequency of eosinophils significantly increased (*p =* 0.0313), and the frequency of neutrophils significantly decreased (*p =* 0.0313). As the frequency of lymphocytes remained stable during this period, this led to a trend for a decrease in the ratio of neutrophils to lymphocytes (*p =* 0.0625). Although the frequency of granulocytes did not significantly change over the first three years of life in the infant macaques cohort, a significantly higher frequency of granulocytes was measured in the group of young adult macaques (12 weeks of age: *p =* 0.0256; 16 weeks of age: *p =* 0.05; 20 weeks of age: *p =* 0.0002; one year: *p =* 0.0023; 3 years of age: *p =* 0.02). The frequencies of lymphocytes and neutrophils remained constant over the first few years of life but there were significantly lower numbers of lymphocytes measured in the group of young adult macaques (close to birth: *p =* 0.0154; one year: *p =* 0.0154; two year: *p =* 0.0247; three years: *p =* 0.0117) of age. This resulted in a significantly higher neutrophil to lymphocyte ratio (N:L) compared to one and three-year-old macaques (*p =* 0.0256). The frequency of eosinophils was also significantly decreased in the group of young adults compared to the levels measured in the infants macaques as they matured (12 weeks of age: *p =* 0.0154; 16 weeks of age: *p =* 0.0256; 20 weeks of age: *p =* 0.0023) but not different from the macaques as they matured from one year to three years (**Figures 6F–I**).

Monocytes were identified by their size and granularity scattering properties using a FSC-A *vs* SSC-A dot plot, to which a gating strategy was applied to exclude B lymphocytes (CD20^+^) and NK cells (CD8^+^). Cells expressing CD14 and HLA-DR were then selected and monocyte sub-populations were differentiated by expression of CD14 and CD16 (**Supplementary Figure 7**). The overall frequency of monocytes did not show any significant changes as the macaques progressed through life (**Figure 6J**).

Although the frequency of monocytes did not change throughout the first five years of life, the significant decrease in lymphocytes (**Figure 6K**) in the young adults caused the monocyte to lymphocyte ratio (M:L) to significantly increase in this age group (12 weeks of age: *p =* 0.0256; 16 weeks of age: *p =* 0.0408; 20 weeks of age: *p =* 0.0154; one year: *p =* 0.0117; 2 years of age: *p =* 0.0046, 3 years of age: *p =* 0.0015). The frequency of classical (CD14^+^) monocytes showed a trend to decrease (*p =* 0.0625) during the first 20 weeks after vaccination, while the frequency of non-classical monocytes, which are involved in T-cell stimulation and inflammatory cytokine release (38), showed a trend (*p =* 0.0625) to increase over the same period of time. The frequency of CD16^+^ non-classical (CD16^+^) monocytes continued to increase (*p =* 0.0313) until the age of two before decreasing as macaques reach adulthood (*p =* 0.0313). Coincident with this, the frequency of classical monocytes continued to decrease (*p =* 0.0313) significantly between the ages of one and two (*p =* 0.0313), until two years of age and then significantly increased as macaques reached age five (compared to at 20 weeks of age: *p =* 0.0408; 2 years of age: *p =* 0.0015; 3 years of age: *p =* 0.0046). Significantly higher frequencies of intermediate (CD14^+^CD16^+^) monocytes were identified in the group of young adult macaques than in the cohort of growing macaques when 12 weeks (*p =* 0.0408), 16 weeks (*p =* 0.0326), 20 weeks (*p =* 0.0256), or three years (*p =* 0.0162) of age. (**Figures 6L–O**).

NK cells were identified by gating on lymphocytes and then excluding doublet cells, dead cells, B lymphocytes (CD20^+^) and monocytes (CD14^+^) after which cells were sequentially gated on their lack of CD3 (CD3-). NK cells were identified by their CD8, CD16 and CD56 expression (**Supplementary** **Figure 7**). Similarly to the monocytes, the total frequency of NK cells did not show any significant changes as the macaques aged, although infant macaques had significantly more CD56^+^ NK cells, typically thought to be cytokine-producing, than macaques aged one year, two years, three years (*p =* 0.0313) and five years (*p =* 0.0426) of age (**Figures 6P–T**). The frequency of T-regulatory cells did not significantly change in the infant cohort during the initial 20 weeks of life, or as they aged to three years, and the frequencies were similar to those measured in the young adult macaque group (data not shown).

**Figure 7:**
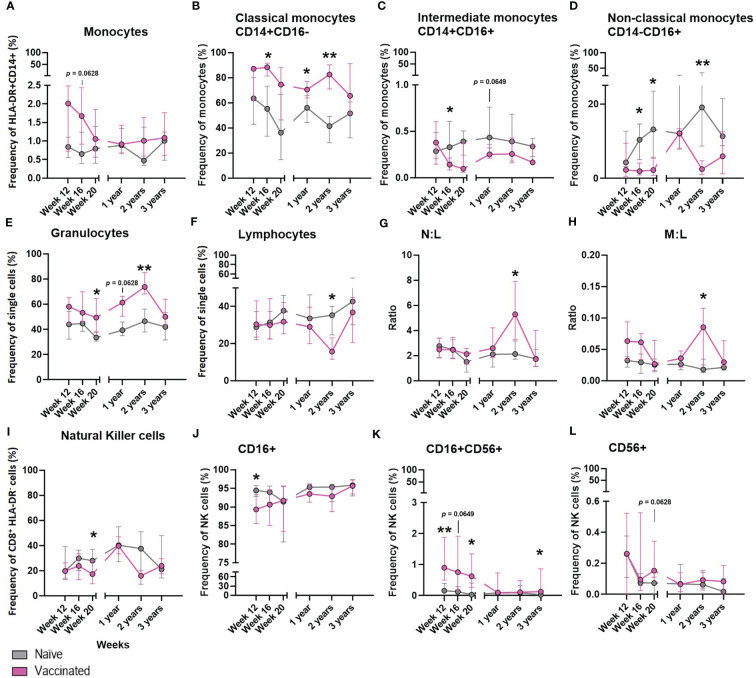
The effect of BCG on the development of whole blood cell populations in neonatal macaques. Plots show the median frequency of **(A)** total monocytes, **(B)** classical monocytes, **(C)** intermediate monocytes, **(D)** non-classical monocytes, **(E)** granulocytes, **(F)** Total lymphocytes, **(G)** neutrophil: lymphocyte ratio (N:L) **(H)** monocyte: lymphocyte ratio (M:L) **(I)** total natural killer cells **(J)** CD16+ NK cells **(K)** CD16+CD56+ NK cells and **(L)** CD56+ NK cells from infancy (12 weeks of age) through to three years in macaques vaccinated within a week of birth (pink) and unvaccinated age-matched controls (grey). Plots show the group median +/- the inter-quartile range. Significant differences measured by Mann-Whitney U-test between the groups at each time point are indicated in black. *p ≤ 0.05, **p < 0.01.

### The Impact of BCG Vaccination on the Innate Immune Cells of Neonatal Macaques

The frequency of cell populations measured in peripheral whole blood in a subset of six BCG vaccinated macaques was monitored at intervals over the first three years of life alongside six unvaccinated age-matched controls to investigate the influence of BCG vaccination on the cellular immune compartment.

There was a non-significant trend (*p =* 0.0628) for a higher frequency of total monocytes to be present in the whole blood of macaques after vaccination with BCG compared to those who did not receive the vaccine (**Figure 7A**). A significantly higher frequency of classical monocytes was measured in the BCG vaccinated infants at week 20 (*p =* 0.0260), one year (*p =* 0.0238) and two years (*p =* 0.0022) of age compared to the level seen in the unvaccinated group. Conversely, naïve macaques possessed a significantly higher frequency of immunomodulatory non-classical monocytes at 16 weeks (*p =* 0.0260), 20 weeks (*p =* 0.0152) and two years (*p =* 0.0022) of age. At 16 weeks of age there was also a significantly higher frequency of intermediate monocytes (*p =* 0.0368) in the unvaccinated macaques which continued at two years of age (*p =* 0.0649) (**Figures 7B–D**).

BCG vaccinated infants possessed a significantly higher frequency of granulocytes (**Figure 7E**) at week 20 (*p =* 0.0152 and two years (*p =* 0.0087), and also a trend at one year (*p =* 0.0649) of age compared to the naïve macaques. The frequency of total lymphocytes (**Figure 7F**) decreased in proportion to the increase of granulocytes in the vaccinated group at two years of age, which was not seen in the naïve macaques. Although differences in the frequency of neutrophils (data not shown) between the vaccinated and unvaccinated animals did not reach significance, the decrease in lymphocytes in the vaccinated macaques accounted for a significantly higher neutrophil to lymphocyte ratio (**Figure 7G**) at two years of age (*p =* 0.0260) and also in the monocyte to lymphocyte ratio (**Figure 7H**).

The frequency of NK cells present in macaques that received BCG close to birth was significantly lower (*p =* 0.0368) 20 weeks after vaccination compared to the unvaccinated controls (**Figure 7I**). Twelve weeks after BCG, vaccinated macaques had a significantly higher frequency of CD16^+^CD56^+^ NK cells present in the periphery (week 12: *p =* 0.0087, week 20: *p =* 0.0260), while the frequency of CD16^+^CD56- NK cells was significantly lower (week 12: *p =* 0.0411). Three years after vaccination, a significantly higher (*p =* 0.0260) frequency of double positive (CD16^+^ CD56^+^) NK cells remained. There was also a trend (*p =* 0.0628) for an increased frequency of CD16-CD56^+^ NK cells in the BCG vaccinated infants 20 weeks after vaccination (**Figures 7J–L**). The frequencies of eosinophils, T-cells, CD4^+^ or CD8^+^ T-cells, activated lymphocytes, B-cells, T-regulatory cells or neutrophils were not changed by administration of the BCG vaccine (data not shown).

To further investigate the effect that age at the time of vaccination had on the immune response to BCG, the quantity of cytokine produced by the total NK and monocyte populations was assessed. Multi-parameter ICS staining was applied to stimulated whole blood collected from the six macaques vaccinated within a week of birth, six unvaccinated infant age matched controls and eight young adult animals. The frequencies of cells producing the cytokines IFN-γ, TNF-α, IL-2 and IL-17 were measured just prior to BCG vaccination (week 0) and at week 12, 16 and 20 weeks after vaccination in the young adult macaque group to provide a direct comparison to the macaques vaccinated within a week of birth. Due to their young age at vaccination, blood sample collection prior to vaccination was not possible for the neonatally-vaccinated group Therefore, the first samples collected from unvaccinated macaques at 12 weeks of age were used to represent the immune responses in infants before vaccination.

**Figure 8:**
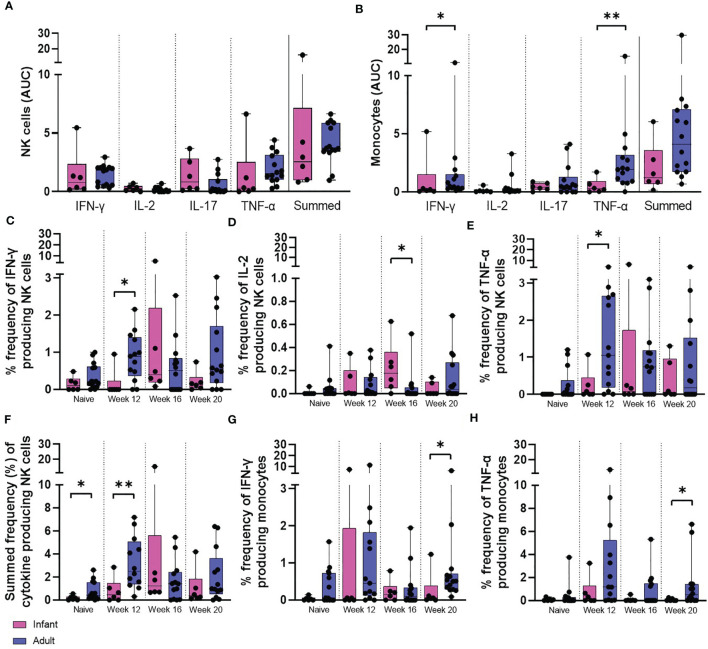
PPD-induced cytokine production by NK cells and monocytes. Total PPD-induced cytokine production by **(A)** NK cells and **(B)** monocytes. Dots represent the AUC of cells producing IFN-γ, IL-2, IL-17, TNF-α and summed frequencies of cytokine producing cells measured in stimulated whole blood collected from individual infant (pink bars) and adult (blue bars) animals between weeks 12 and 20. Plots show the frequency of **(C)** IFN-γ, **(D)** IL-2, **(E)** TNF-α and **(F)** Total summed cytokine produced by NK cells and **(G)** IFN-γ and **(H)** TNF-α produced by monocytes prior to and following ID BCG vaccination. Box plots show the group median +/- the inter-quartile range, with minimum and maximum values connected by whiskers. Significant differences measured by Mann-Whitney U-test between the groups are indicated. *p ≤ 0.05, **p < 0.01.

The frequency of NK cells producing IFN-γ (*p =* 0.0154) and TNF-α (*p =* 0.0369) was significantly higher at 12 weeks after vaccination in the adult vaccinated group compared to the infant vaccinated group. Macaques vaccinated shortly after birth possessed a significantly higher frequency of NK cells producing IL-2 (*p =* 0.0168) 16 weeks after vaccination compared to those vaccinated as adults. Comparison of summed frequencies of cytokine producing NK cells collected 12 weeks after vaccination from adult macaques produced significantly more cytokine than those from the cohort vaccinated as infants (*p =* 0.0094). However, area under the curve analysis (AUC) of the cytokine producing cells during the vaccination phase (12 to 20 weeks following vaccination) indicated equivalence between the two age groups (**Figures 8A, C–F**).

The frequency of functional monocytes producing IFN-γ (*p =* 0.0154) or TNF-α 20 weeks after vaccination (*p =* 0.0361) was significantly higher in the adult vaccinated group in concordance with the findings of area under the curve analysis (AUC) of the vaccination phase (IFN-γ: *p =* 0.05, TNF-α: *p =* 0.0064) (**Figures 8B, G, H**).

## Discussion

In endemic regions, BCG is given to infants near to birth, but most pre-clinical TB vaccine studies use adult animals (26). Understanding the host response to BCG vaccination in both a mature and an immature immune system is essential to understand how BCG and new vaccine strategies can induce immunity and provide protection. This knowledge will also provide insight into the most appropriate pre-clinical models to use for the assessment of vaccines designed to work in infants.

The systemic cellular immune response to BCG delivered intradermally in macaques that have reached adulthood has previously been described (10, 11). In this study, we set out to define the host immune status of neonatal macaques as they mature and to evaluate the immunological response induced in rhesus macaques when BCG is delivered within a week of birth to further our understanding of how BCG shapes the immune system in the initial weeks after vaccination and also long term, and also how the infant is able to respond with an immature immune system.

As shown by the increased frequency of IFN-γ secreting cells and the levels of cytokines and chemokines quantified by multiplex bead array, BCG-specific immune responses were elicited following vaccination in macaques vaccinated within a week of birth. This indicates that although infant macaques are documented to have a ‘naïve’ immune system with many reporting dampened inflammatory responses (16, 22, 39), these cells are able to generate a functional response to the vaccine. However, these responses are lower than responses typically observed in BCG vaccinated adult macaques. This reduced immunogenicity is consistent with profiles measured in BCG vaccinated human populations (26) where BCG is known to provide protection from severe forms of childhood disease. It may be hypothesized that the naïve immune system allows the BCG vaccine to persist and continuously stimulate the immune system to provide protection but is eventually cleared by the immune cells present as an adult which leads to the waning of protection (40).

To understand the response induced by BCG when delivered in infancy, we characterised the host cell immune status as macaques developed from six to 12 weeks after birth, through to three years, and also compared to young adult macaques aged between 3.7 and 5.2 years of age. As previously shown (28, 41), the frequency of immune cells changed as the infants developed through the first few years of life, and in comparison to immune cell frequencies measured in a separate group of young adult macaques. Notably, the frequency of both CD4^+^ and CD8^+^ T-cells expressing memory marker CD95 increased as the macaques aged. This is likely to be a consequence of accumulated antigen exposure as the macaque’s immune system encounters environmental antigens and pathogens which drives T-cell maturation and promotes a significant increase in the frequency of CD95^+^ T-cells over time. The significantly lower production of pro-inflammatory cytokines such as IFN-γ, TNF-α, IL-2 and IL-6, measured using ELISPOT, multiplex bead array and intracellular staining assays, seen in the vaccinated infants compared to that of an immunologically mature adult may be due to the infant immune system comprised predominantly of naïve immune cells. It is widely believed that BCG predominantly mediates immunity by the development of antigen-specific memory T cells (42), and naïve cells (CD95-) are unable to respond to antigen by producing cytokine. Therefore, it is perhaps unsurprising that BCG given to infants within a week of birth generated a lower magnitude of response when compared to similarly vaccinated adult macaques.

The effect of BCG delivered in infancy on the cellular immune compartment may manifest overtime, for example, a significant decrease in the lymphocyte population which affected both the N:L and M:L ratios was observed, together with a significantly higher frequency of CD8+ TTE cells in the macaques two years after vaccination. Age related changes independent of BCG vaccination were also observed as the macaques reached two years old, B-cells were found to significantly increase while T cells decreased. As these changes coincided with the point at which the macaques were weaned from their mother, it suggests that external life factors may also influence the composition of the immune system and contribute to inflammatory effects of the vaccine making this an area that requires further investigation.

The concentration of chemokine markers MIP-1α, MIP-1β and MIG, typically produced by macrophages and monocytes after stimulation, were significantly increased in macaques vaccinated in infancy compared to unvaccinated age-matched controls. These markers are considered important for responses to fight infection and control inflammation. These chemokines affect monocytes, T-cells, dendritic cells, NK cells, activate granulocytes and induce the synthesis and release of pro-inflammatory cytokines such as IL-1, IL-6 and TNF-α from macrophages (43, 44) which were also significantly increased in the vaccinated infants. Data from the immunophenotyping also showed that BCG vaccination significantly affected the frequency of the monocyte, granulocyte and NK cell subsets in infants which is indicative of an innate-like, trained immune response being induced by BCG, as previously reported in adults (24).

Classical monocytes are known for their antigen presenting and phagocytic abilities, which may be responsible for the increase in the frequency of this population in the macaques vaccinated in infancy. There is evidence that BCG causes long term non-specific effects on the host immune system including increased expression of pattern recognition receptors such as CD14 (45) which can be seen in these data. The M:L ratio was found to be significantly higher in young adult macaques compared to macaques aged between 12 weeks and three years of age. As an elevated M:L ratio has been associated with the risk of subsequent tuberculosis disease (46), this may suggest the young adults have an increased risk of developing disease compared to infant macaques.

BCG vaccination of infant humans induces activation of innate effector cells such as NK cells (24). Here we found that cytokine producing CD56^+^ NK cells were present at a significantly higher frequency in 12 week old macaques compared to young adults and these were significantly increased after vaccination in infant macaques. CD56^+^ NK cell subsets have a higher capacity to produce cytokines following activation by monocytes which suggests that a trained immunity phenotype, characterized by an increased capacity to produce proinflammatory cytokines, is being induced in infants when vaccinated shortly after birth.

There was also significantly more antigen-specific production of the modulatory cytokines IL-1RA and SCF in the infant BCG vaccinated group. IL-1RA inhibits and modulates IL-1 responses and SCF is also a pro-inflammatory inhibitor secreted by mast cells to suppress the immune response including the suppression of TNF-α and IL-6. This pattern of inhibitory cytokine secretion is different to the cytokine profile measured in vaccinated adult macaques where significantly increased production of Th2 cytokines such as IL-4, IL-5 and IL-15 was detected, suggesting regulation of the proinflammatory response in a different manner.

This study demonstrates multiple differences between infant rhesus macaques aged between six and 12 weeks of age and young adult macaques aged between 3.7 and 5.2 years of age. These differences correspond with distinct responses to the BCG vaccine. As most animal models currently used to study TB vaccine candidates rely on adult animals, which in our study produced significantly higher amounts of cytokine following BCG vaccination compared to macaques vaccinated in infancy, understanding the immune responses generated when BCG is given early in life is essential. Further investigation needs to be done using neonatal and age appropriate models to understand the protective efficacy afforded by the BCG vaccine and for the development of improved vaccination strategies for deployment in infants.

BCG provides protection to infants against severe forms of TB, has been shown to give protection against other non-TB pathogens, is associated with lower mortality in children, and can improve the efficacy of other childhood vaccines (47). However, it is widely believed that BCG protection is not durable past adolescence (48). Our data show that BCG alters the frequencies of classical monocytes, CD56^+^ NK cells and granulocytes present in the periphery for at least three years after vaccination. Although this change in host cell frequencies may not be enough to provide continued protection against *M. tuberculosis*, it may alter the immune response to booster vaccinations. This needs to be considered when developing improved vaccination strategies to understand whether BCG will enhance, or mask, additional vaccinations given in later life. Further studies investigating the effect that age at vaccination has on the protective efficacy afforded by BCG as well as additional vaccines are needed.

## Data Availability Statement

The original contributions presented in the study are included in the article/**Supplementary Material**. Further inquiries can be directed to the corresponding author.

## Ethics Statement

The animal study was reviewed and approved by UK Health Security Agency, Animal Welfare and Ethical Review Body, Porton Down, UK, and authorized under an appropriate UK Home Office project license.

## Author Contributions

CS, SS, AW, and LS contributed to the conceptualization and methodology. CS, LS, AM, and JG performed experiments. MD and SL performed sample collection and animal expertise. CS performed analysis. AW, SS, PM, and HF provided supervision. CS wrote the paper and all authors provided assistance and critical review. All authors contributed to the article and approved the submitted version.

## Funding

This work was support by funding awarded from Aeras Global Tb Foundation, EU Commission H2020 project 730964 TRANSVAC2, UK Department of Health.

## Conflict of Interest

The authors declare that the research was conducted in the absence of any commercial or financial relationships that could be construed as a potential conflict of interest.

## Publisher’s Note

All claims expressed in this article are solely those of the authors and do not necessarily represent those of their affiliated organizations, or those of the publisher, the editors and the reviewers. Any product that may be evaluated in this article, or claim that may be made by its manufacturer, is not guaranteed or endorsed by the publisher.
