# Supplementary material for: Characterization of the Infant Immune System and the Influence and Immunogenicity of BCG Vaccination in Infant and Adult Rhesus Macaques
Source: Front Immunol. 2021 Oct 11;12:754589. doi: 10.3389/fimmu.2021.754589 (PMC8542880; doi:10.3389/fimmu.2021.754589)
Supplement: Supplementary file 1 [file DataSheet_1.pdf]

Supplementary data

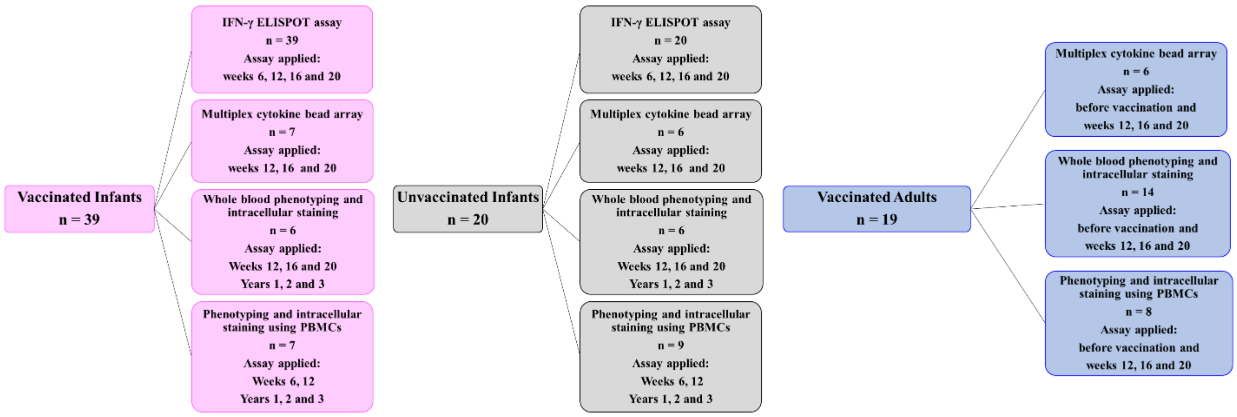

Supplementary Figure 1: Immunological analysis. Figure describes the immunological analyses applied to samples collected from each study cohort of macaques. Due to small volumes of blood permitted for collection from the infant macaques, animals were randomly assigned to sub-groups to enable completion of each type of immunological analysis.

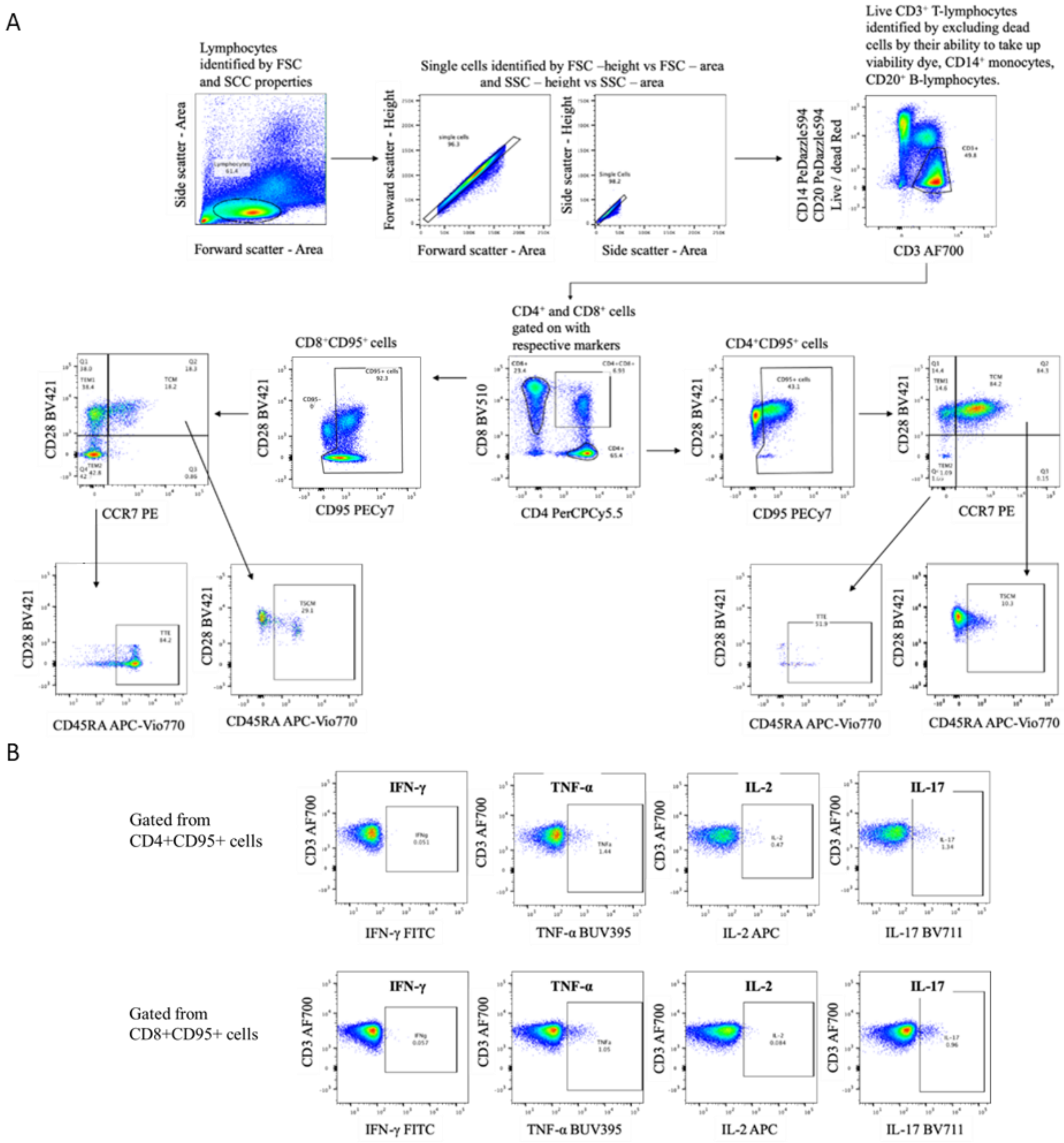

Supplementary Figure 2. Flow cytometric analysis of T-Lymphocyte and memory cell subsets in PBMCs. (A) $CD4^+$, $CD8^+$ and $CD4^+CD8^+$ T-cells were identified by first gating on FSC (forward scatter) vs SSC (side scatter) to identify lymphocytes, then FSC height vs area followed by SSC height vs area to identify single cell events. Live $CD3^+$ T-lymphocytes were identified through exclusion of $CD20^+$ B-lymphocytes, $CD14^+$ monocytes and non-viable dead cells. $CD4^+$, $CD8^+$ and $CD4^+CD8^+$ cells were identified using respective markers. Memory cells were identified by gating on activated CD4 and CD8 T-cells using CD95 staining. Memory T-cell populations were differentiated by their expression of CD28, CCR7 and CD45RA. (B) Cytokine producing cells were identified by gating on the $CD95^+$ populations followed by gating on cells that stained positively for the production of IFN-γ, TNF-α, IL-2 or IL-17.

Memory cell subtype	Age group		P value
**CD4$^+$**	6 weeks	12 weeks	NS
		1 year	*0.0156
		2 years	0.0625
		3 years	**0.0078
		4 - 5 years	***0.0002
	12 weeks	1 year	**0.0039
		2 years	*0.0313
		3 years	**0.0039
		4 - 5 years	****<0.0001
	1 years	2 years	NS
		3 years	NS
		4 - 5 years	**0.0017
	2 years	3 years	NS
		4 - 5 years	*0.0426
	3 years	4 - 5 years	*0.0164
**CD8$^+$**	6 weeks	12 weeks	NS
		1 year	**0.0078
		2 years	0.0625
		3 years	**0.0078
		4 - 5 years	***0.0002
	12 weeks	1 year	**0.0039
		2 years	*0.0313
		3 years	**0.0039
		4 - 5 years	****<0.0001
	1 year	2 years	NS
		3 years	NS
		4 - 5 years	***0.0010
	2 years	3 years	NS
		4 - 5 years	**0.0080
	3 years	4 - 5 years	*0.0274
**CD4$^+$ CD95$^+$**	6 weeks	12 weeks	*0.0391
		1 year	**0.0078
		2 years	0.0625
		3 years	**0.0078
		4 - 5 years	***0.0002
	12 weeks	1 year	**0.0039
		2 years	*0.0313
		3 years	**0.0039
		4 - 5 years	<0.0001
	1 years	2 years	*0.0313
		3 years	*0.0195
		4 - 5 years	***0.0003
	2 years	3 years	NS
		4 - 5 years	NS
	3 years	4 - 5 years	NS
**CD8$^+$ CD95$^+$**	6 weeks	12 weeks	NS
		1 year	**0.0078
		2 years	NS
		3 years	**0.0078
		4 - 5 years	***0.0002
	12 weeks	1 year	**0.0039
		2 years	*0.0313
		3 years	**0.0039
		4 - 5 years	****<0.0001
	1 years	2 years	NS
		3 years	NS
		4 - 5 years	**0.0035
	2 years	3 years	NS
		4 - 5 years	NS
	3 years	4 - 5 years	*0.0152

Supplementary Table 1. P values resulting from the statistical comparison of CD4$^+$ and CD8$^+$ T-cell and CD4$^+$CD95$^+$ and CD8$^+$CD95$^+$ memory population numbers determined during the first three years of life, and in comparison with the numbers in young adult macaques. Differences were evaluated using a Wilcoxon matched-pairs rank test within the group or Mann-Whitney U-test between the groups. Significant differences are indicated. * $p < 0.05$, ** $p < 0.01$, *** $p < 0.001$, **** $p < 0.0001$.

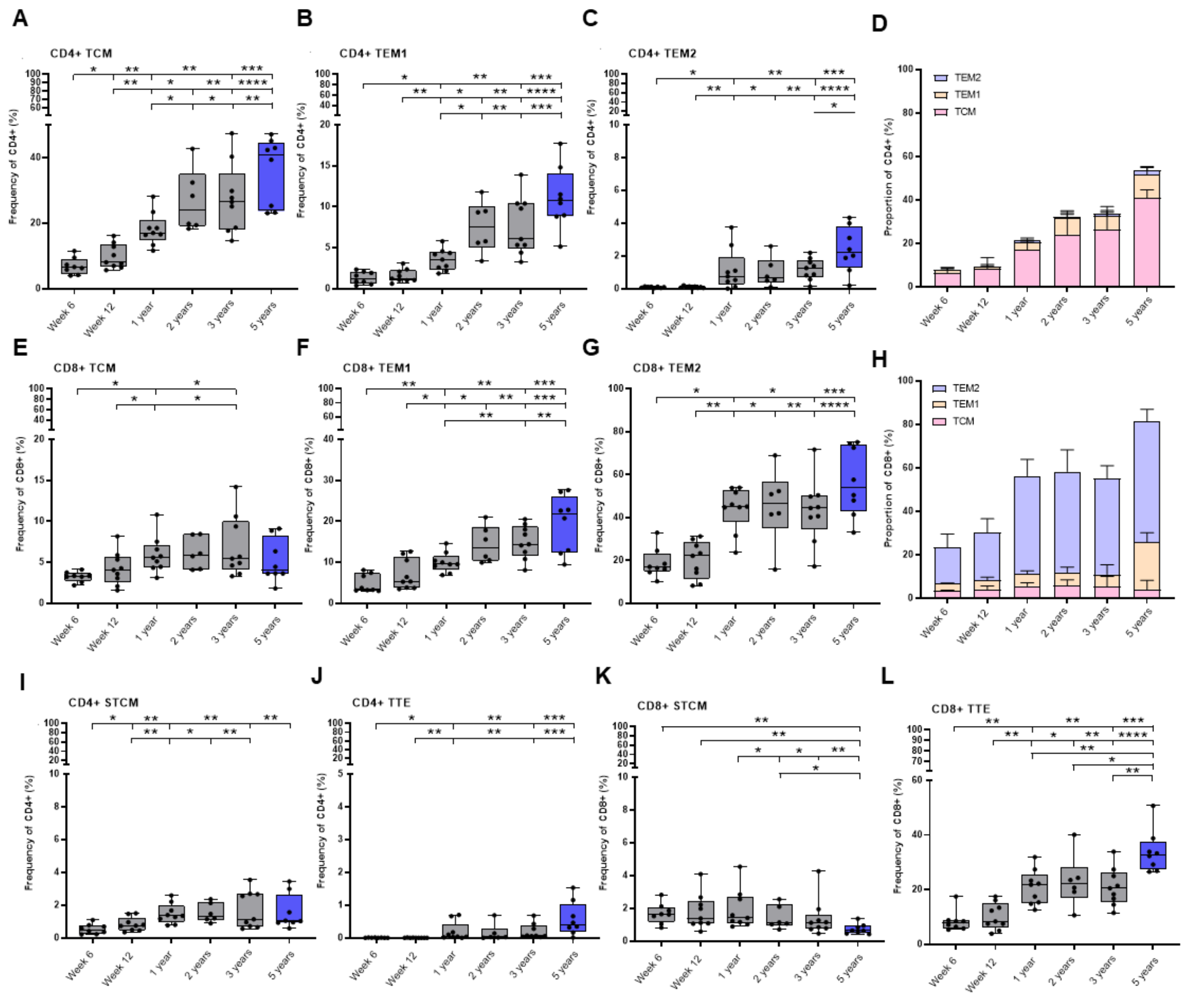

Supplementary Figure 3. Age related changes in memory cell subset populations. Plots (A) to (F) show the frequency of CD4^{+} and CD8^{+} effector and central memory cell subsets, (G) to (H) proportions of TEM1, TEM2 and TCM and (I) to (L) frequency of CD4^{+} and CD8^{+} STCM and TTE measured in naïve macaques from infancy (6 weeks of age) through to three years (grey) compared with young adult macaques aged between 3.7 and 5.2 years of age (blue). Box plots show the group median +/- the inter-quartile range, with minimum and maximum values connected by whiskers. Plots show the group median +/- the inter-quartile range. Significant differences measured by Wilcoxon matched-pairs rank test or Mann-Whitney U-test between the groups are indicated. * $p < 0.05$, ** $p < 0.01$, *** $p < 0.001$, **** $p < 0.0001$

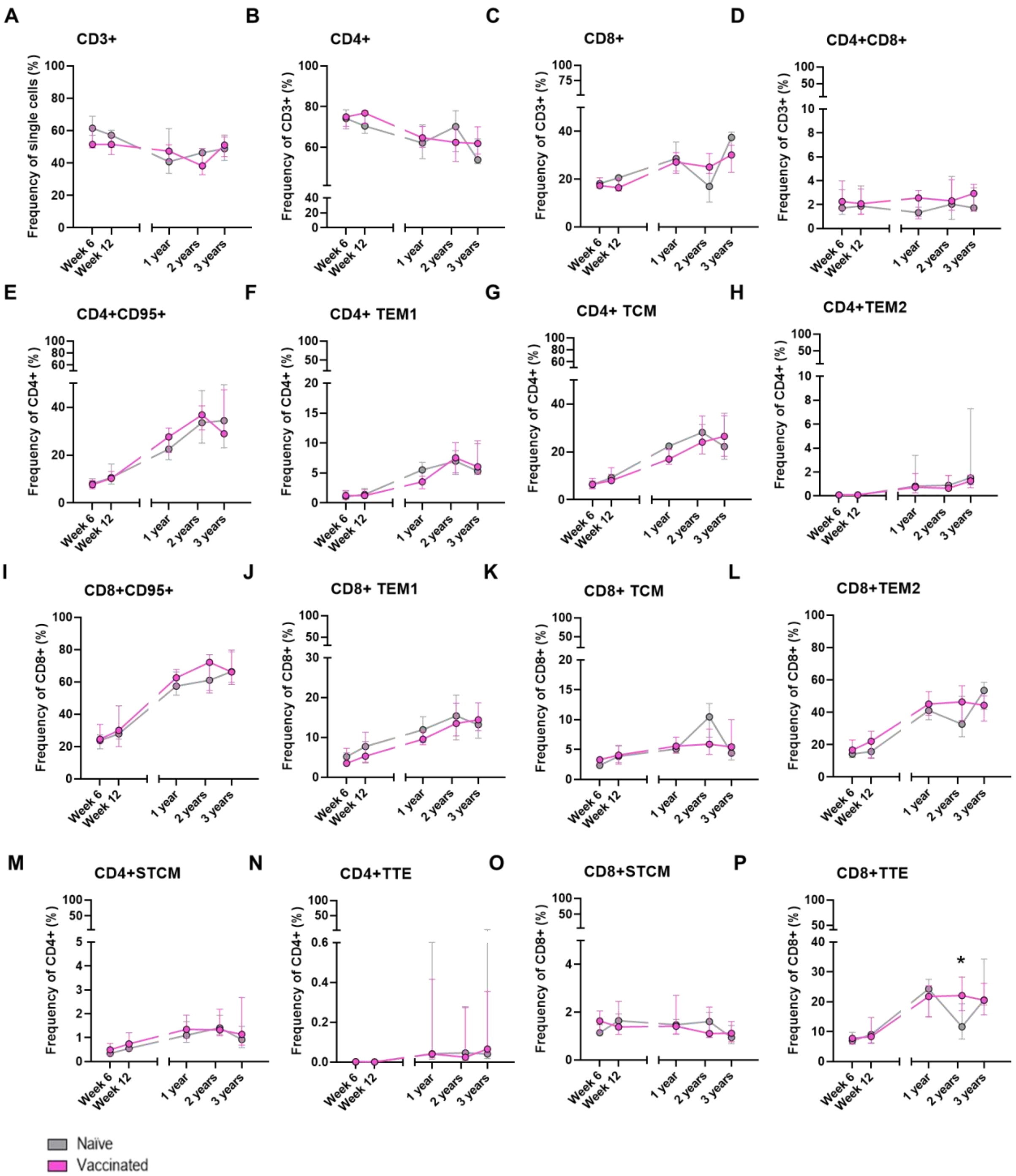

Supplementary Figure 4. The effect of BCG on the evolution of lymphocytes and T-cell memory subset populations. Plots show the median frequency of (A) CD3^{+}, (B) CD4^{+}, (C) CD8^{+}, (D) CD4^{+}CD8^{+}, (E) CD4^{+}CD95^{+}, (F) CD4^{+}TEM1, (G) CD4^{+}TCM (H) CD4^{+}TEM2 (I) CD8^{+}CD95^{+} (J) CD8^{+}TEM1 (K) CD8^{+}TCM (L) CD4^{+}TEM2, (M) CD4^{+}STCM (N) CD4^{+}TTE , (O) CD8^{+}STCM and (P) CD8^{+}TTE from infancy (12 weeks of age) through to three years in macaques vaccinated within a week of birth (pink) and unvaccinated age-matched controls (grey). Plots show the group median +/- the inter-quartile range. Significant differences measured by Mann-Whitney U-test between the groups at each time point are indicated in black. * $p \leq 0.05$.

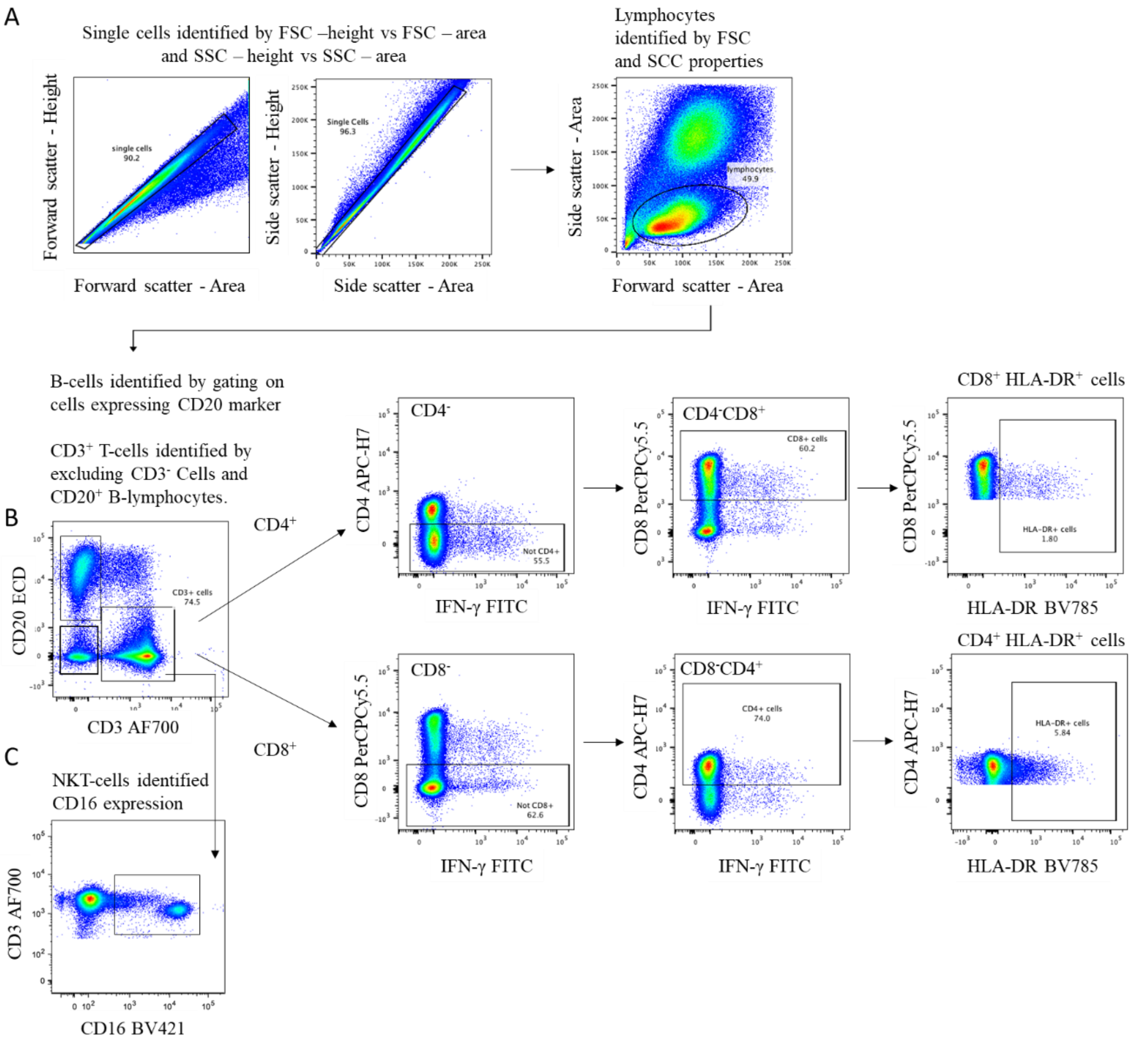

Supplementary Figure 5. Flow cytometric analysis of activated T-Lymphocytes, B-lymphocytes and NKT-lymphocytes in whole blood. (A) Single cell events were identified using a FSC height vs area gate followed by SSC height vs area gate. Activated CD4$^+$ and CD8$^+$ T-cells were identified with an initial FSC (forward scatter) vs SSC (side scatter) gate to identify lymphocytes, then (B) CD3$^+$ T-lymphocytes were identified by exclusion of CD20$^+$ B-lymphocytes. CD4$^+$ and CD8$^+$ T-cells were distinguished by identification of CD8- followed by CD4$^+$ subsets (or the reciprocal staining pattern for identification of CD8$^+$ subsets), followed by gating on cells positively stained for HLA-DR. CD20+ B-lymphocytes were identified by gating on CD20$^+$ cells and excluding CD3$^+$ Cells. (C) NKT-cells were identified from the CD3$^+$ population by gating on CD16$^+$ cells.

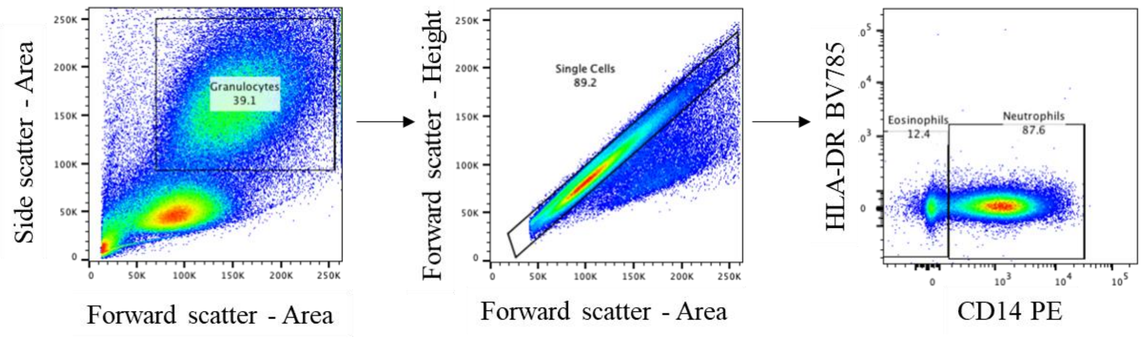

Supplementary Figure 6. Flow cytometric analysis of granulocyte populations. Granulocytes were identified using a FSC (forward scatter) vs SSC (side scatter) gate, from which single events were identified by gating on FSC height vs area Eosinophils and Neutrophils were identified by their lack of HLA-DR expression and differentiated by CD14 expression.

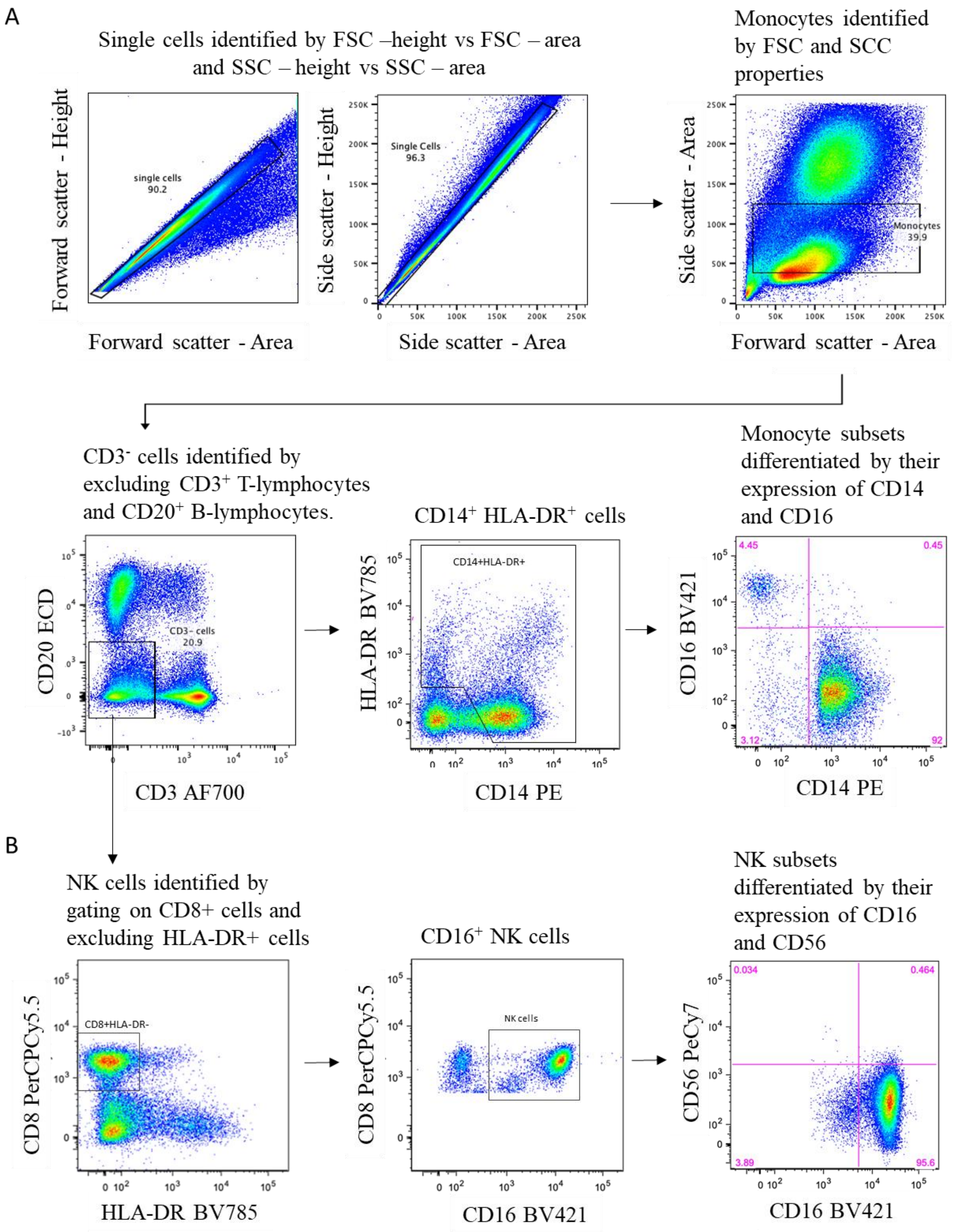

Supplementary Figure 7. Flow cytometric analysis of monocyte subset populations by differential expression of CD14 and CD16. Cells were gated on FSC height vs area followed by SSC height vs area to identify single cell events to identify single cell events. (A) Monocytes were identified by first gating on FSC (forward scatter) vs SSC (side scatter) then $CD20^+$ B-lymphocytes and $CD3^+$ T-lymphocytes were excluded. Monocytes were identified as cells expressing CD14 and HLA-DR and monocyte subsets were differentiated by expression of CD14 and CD16. (B) NK cells were identified from the $CD3^-$ population by their expression of CD8 and lack of expression of HLA-DR. Following this, NK cells were selected for presentation of CD16 marker. NK cell subsets were differentiated by expression of CD16 and CD56.
